# Comparison of Experiences in Two Birth Cohorts Comprising Young Families with Children under Four Years during the Initial COVID-19 Lockdown in Australia and the UK: A Qualitative Study

**DOI:** 10.3390/ijerph18179119

**Published:** 2021-08-29

**Authors:** Lisa Y. Gibson, Bridget Lockyer, Josie Dickerson, Charlotte Endacott, Sally Bridges, Rosemary R. C. McEachan, Kate E. Pickett, Sarah Whalan, Natasha L. Bear, Desiree T. Silva, Susan L. Prescott, Jacqueline A. Davis

**Affiliations:** 1 Telethon Kids Institute, 15 Hospital Avenue, Nedlands, WA 6009, Australia; Sarah.Whalan@telethonkids.org.au (S.W.); desiree.silva@telethonkids.org.au (D.T.S.); Susan.Prescott@telethonkids.org.au (S.L.P.); Jackie.Davis@telethonkids.org.au (J.A.D.); 2 School of Medicine, The University of Western Australia, Perth, WA 6009, Australia; 3 VIVO Planetary Health, Worldwide Universities Network (WUNWest), New York, NY 10704, USA; 4 School of Medicine & Health Sciences, Edith Cowan University, Perth, WA 6027, Australia; 5 Bradford Institute for Health Research, Bradford Teaching Hospitals NHS Foundation Trust, Bradford BD9 6RJ, UK; Bridget.Lockyer@bthft.nhs.uk (B.L.); Josie.Dickerson@bthft.nhs.uk (J.D.); charlotte.endacott@bthft.nhs.uk (C.E.); Sally.Bridges@bthft.nhs.uk (S.B.); rosie.mceachan@bthft.nhs.uk (R.R.C.M.); 6 Department of Health Sciences, University of York, York YO10 5DD, UK; kate.pickett@york.ac.uk; 7 Institute for Health Research, Notre Dame University, Fremantle, WA 6160, Australia; natasha@bearstats.com.au; 8 School of Public Health, Curtin University, Bentley, WA 6102, Australia; 9 Joondalup Health Campus, Joondalup, WA 6027, Australia; 10 Perth Children’s Hospital, 15 Hospital Avenue, Nedlands, WA 6009, Australia; 11 NOVA Institute for Health of People, Places and Planet, Baltimore, MD 21231, USA; 12 Department of Family and Community Medicine, University of Maryland School of Medicine, Baltimore, MD 21201, USA

**Keywords:** COVID-19, cohort study, wellbeing, qualitative data, worries, challenges, anxiety, families, collaborative research

## Abstract

This study aims to understand the experience and impact of the initial COVID-19 lockdown in young families with children aged below 4 years. Free text questions were administered to participants in the ORIGINS (Australia) and Born in Bradford (UK) cohort studies to collect qualitative information on worries, concerns and enjoyable experiences during the pandemic. A total of 903 (400 for ORIGINS and 503 for BiB) participants completed the two surveys during April 2020. Despite varying in geography, levels of socio-economic disadvantage and their situational context during the pandemic, respondents from both cohorts reported similar worries and challenges during the lockdown period, including: employment/finances, health anxiety, mental health and social isolation, caring for children and child development. Families across the globe experienced both positive and negative immediate impacts of COVID-19. Population-based data can be used to inform the development of support services, public health campaigns and universal interventions to assist families in future health crises.

## 1. Introduction

On 11 March 2020, The World Health Organisation (WHO) declared COVID-19 a pandemic. Shortly thereafter, on 23 March 2020, the UK (United Kingdom) and Australian governments, along with many other nations, implemented stringent lockdowns to stop the spread of the virus. This included the closure of schools, non-essential shops and businesses, reduced health and social care provision and restrictions on daily activities. These measures were largely aimed at limiting viral transmission, the number of severe cases, COVID-19 deaths, and consequent pressures on health care systems [1].

While the focus of these policies has been on implementing strategies to slow the rate of infection, there is increasing concern surrounding the wider impacts of the fear of the pandemic and the restrictive lockdowns on the general population, including children and young families who may be less vulnerable to the infection, but no less vulnerable to the effects of fear and isolation. Research suggests that disease outbreaks can have severe impacts on mental health and wellbeing, as reported previously during epidemics of SARS [2], the MERS, H1N1 and Ebola [3]. However, the sheer scale of the COVID-19 pandemic on global mortality and morbidity has far exceeded that of any recent disease outbreaks, raising more significant acute and long-term concerns. Furthermore, the large-scale impact of lockdown restrictions on social, emotional and economic well-being are predicted to have unprecedented and extensive implications for mental health in populations more broadly, independent of biological effects of infection [4]. 

The additional dimension of impact on mental wellbeing conferred by pandemic-associated restrictions in the broader community had been referred to as the “second pandemic”. Already, international data have revealed the acute impact of the pandemic on the general population, with increased feelings of stress, anxiety, depression, insomnia, denial, anger and fear [5,6,7]. More significant symptoms of moderate to severe depression, anxiety and levels of stress have also been reported across both developed and developing countries [8,9,10,11,12,13]. However, the impacts are not experienced equally across societies. Higher levels of depression, anxiety and stress are being observed in individuals with low levels of education and low socio-economic status in many regions [8,10,14]. It is predicted that the ongoing, longer-term social and emotional impact of the pandemic will place an enormous burden on healthcare systems, social support systems [7] and many other aspects of societies in general. There is a pressing need to understand the short and long term effects of social isolation, lockdown restrictions, loss of employment/income and changes to lifestyle on families and communities [15]. 

Longitudinal family cohort studies provide a valuable window of opportunity to assess the impact of the pandemic on families and communities more fully and answer this urgent call. This will better inform practitioners, policymakers and the communities themselves to guide future health and social care planning. Longitudinal cohort studies are poised for this crucial role in this through the monitoring of long-term health and wellbeing of parents and children—adding the much needed life course perspective as we consider wellness promotion and disease prevention in a vastly changing global landscape. 

We are in a unique position to explore these issues in two birth cohorts—one in the United Kingdom where the impact of COVID-19 infection has been more profound, and another in Western Australia where the community infection has been relatively low. The ORIGINS Project (ORIGINS) is a decade-long collaborative initiative of 10,000 Western Australian (WA) families, enrolled during pregnancy and followed over the first five years of life [16]. In general, these families have above average socio-economic advantage. In contrast, the Born in Bradford (BiB) programme in North of England is a more ethnically diverse population with high levels of deprivation and health inequalities. Based in the fifth largest metropolitan district in England, BiB has collected the health and wellbeing data of over 36,000 Bradford residents since 2007 [17]. 

The existing collaboration between our cohorts provided a valuable opportunity to assess the effects of the global pandemic in these different settings, based on survey data collected in each centre, beginning in April 2020. Both studies collected survey data on their cohort families to understand their experience and the impact of the pandemic on stress, mental health, wellbeing, family functioning and financial hardship. Here, by aligning the open-ended questions between the two cohorts, we report and compare the experiences of young families in two distinct international centres living with the immediate effects and uncertainties of this global crisis. 

## 2. Materials and Methods

Both studies, described in more detail separately below, collected survey data on their cohort families to understand the experience of living through the COVID-19 pandemic and the impact of the pandemic on wellbeing, family functioning and financial hardship. Open-ended questions were aligned between the two cohorts to facilitate direct comparison of families’ experiences in the two populations. 

### 2.1. ORIGINS Project–COVID-19 Data Collection

#### 2.1.1. Setting

Participants enrolled in The ORIGINS Project living in the Joondalup/Wanneroo region in Western Australia were invited to complete the survey assessing the experience of living through the COVID-19 pandemic and the impact of the pandemic on their stress, mental health, wellbeing, family functioning and financial hardship. The sample invited were participants from the ORIGINS cohort (*n* = 2267) which includes families expecting a child as well as families with children 0–4 years old. A total of 461 (20%) completed the survey between 21st April and 5th May, of whom 400 were mothers. 

#### 2.1.2. Ethics

This project has ethical approval from the Ramsay Health Care WA I SA Human Research Ethics Committee (#1440). Prior to the commencement of the online survey, information explaining the purpose and procedures of the study was provided, followed by a consent statement outlining the implications, risks and benefits of participation. Participants were prompted to select a check box (YES/NO) indicating their consent to participate in this study.

#### 2.1.3. Data Collection

Each participant enrolled in The ORIGINS Project was invited via email to complete the online questionnaire. Participants were given a two-week window to complete the survey and were sent a text message reminder to complete the survey at 1 week. As part of the overall project, the same survey was sent to families participating in ORIGINS monthly for four months between April and July 2020. Completing the survey was optional, and participants may have chosen to complete each of the monthly surveys or only one or two of these surveys. 

#### 2.1.4. Measures

Qualitative Questions. A behavioural questionnaire was developed to obtain demographic and behavioural questions related to employment, social distancing and isolation, lifestyle behaviours and engagement with information about COVID-19. The survey also captured qualitative information on worries (“What are your three biggest worries right now?”), challenges (“Can you tell us about a challenge you have faced in the last two weeks?”) and enjoyable experiences (“Can you tell us how lockdown has made any parts of your life easier or more enjoyable?”) during the pandemic in the form of open-end questions and text boxes. 

### 2.2. BiB Project–COVID-19 Data Collection

#### 2.2.1. Setting

A sub-sample of participants in two ongoing BiB cohort studies that had completed recent pre-COVID-19 data collection were invited to complete a questionnaire assessing the experiences of the COVID-19 first lockdown on physical and mental health, financials, food and housing and employment insecurity. The sample invited were from the original BiB cohort of families with children aged 9–13 (N = 5154) and the Born in Bradford’s Better Start (BiBBS) cohort of families with children aged 0–4 (N = 2665). A total of 2144 (28%) completed the survey between April 10th and 30th June 2020, of whom 2043 were mothers [13]. 

#### 2.2.2. Ethics

The research was approved by substantial amendments by the HRA and Bradford/Leeds research ethics committee (BiB Growing Up study 16/YH/0320; BiBBS study 15/YH/0455).

#### 2.2.3. Data Collection 

Participants were asked to complete a single questionnaire during the first COVID-19 lockdown. They were contacted using multiple methods, including a combination of emails, text and phone with a follow-up postal survey in order to facilitate a rapid response. Participants were recruited in their main language wherever possible. 

#### 2.2.4. Measures

The BiB COVID-19 questionnaire is available online [17]. Key domains included: household circumstances; family relationships; loneliness and social support; financial, food, housing and employment insecurity; physical health and mental health. Free text questions asking about the main worries, challenges and positive aspects of lockdown were the same as those asked in the ORIGINS questionnaire.

### 2.3. Comparison of ORIGINS and BiB Findings

Responses to the free text questions from both the ORIGINS and BiB COVID-19 surveys during April 2020 were compared. We chose to only compare the responses from April because after April the ORIGINS survey was repeated monthly with the same participants, whereas the BiB survey data continued to be collected with different participants between April and June. Isolating the April respondents from both surveys was therefore the best way to have the most direct comparison. The ORIGINS cohort only includes pregnant women and families with children 0–4 years old, whereas the BiB cohorts include families with children aged up to 13. As such, only the responses from families with children under 4 from the BiB survey were compared to the ORIGINS responses. 

The demographic characteristics of participants who responded during April 2020 were compared across the two cohorts. Free text responses were analysed separately by the ORIGINS and BiBS teams using NVivio and Excel, respectively, using the principles of reflective thematic analysis [18]. Within the separate teams, the same data was coded by multiple researchers to reduce bias. For both teams, the process of analysis was inductive and was not structured on any existing theoretical frameworks. As a result, the two codebooks developed diverged in format and exact wording, but were, we found, comparable in content and meaning. For example, the ORIGINS team used the code ‘financial concerns’, whereas the BiB team used ‘money worries’ for the same/very similar responses. The two teams shared their analysis and met (virtually) multiple times to identify commonalities and differences in the codebooks and in the content and tone of the responses. In these analysis meetings, we would review samples of both teams’ anonymised data to ensure consistency and reliability between them, following an iterative process of collaborative data analysis described by Hall et al. [19]. Discussions within these meetings also centred on which responses were more or less common within our two data sets, so that we could prioritise the reporting of themes. 

**Figure 1:**
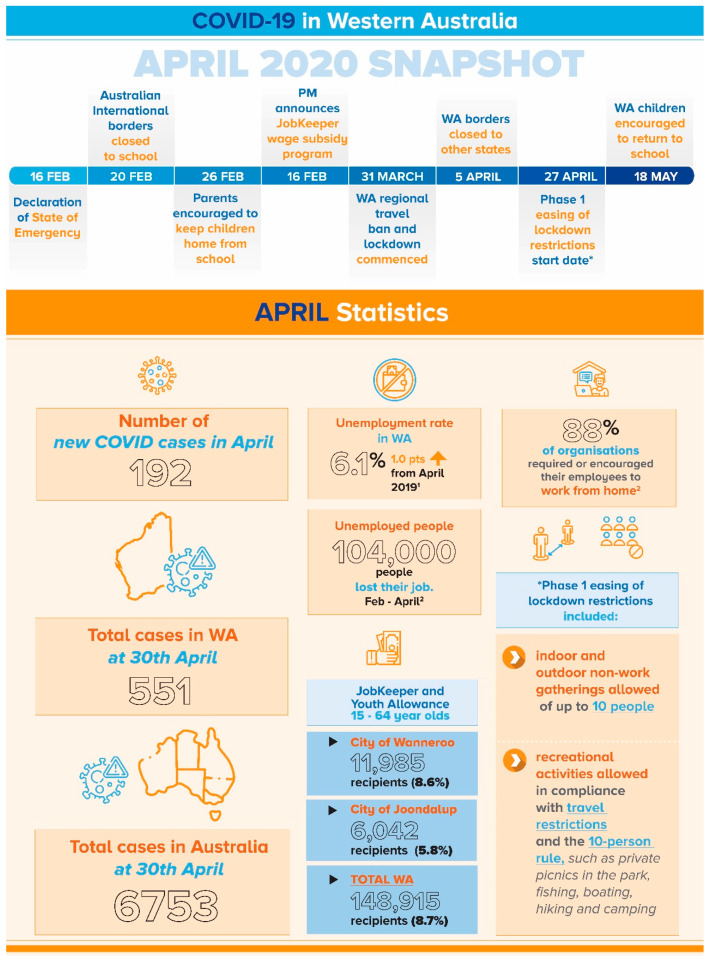
Timeline and Snapshot of COVID-19 events in Perth, Western Australia, as they affected the ORIGINS participants. **Sources:** ^1^ https://www.abs.gov.au/statistics/labour/employment-and-unemployment/labour-force-australia/apr-2020#covid-19-impacts-and-changes; ^2^ https://www.cmo.com.au/article/672072/report-most-australian-employees-work-from-home/.

**Figure 2:**
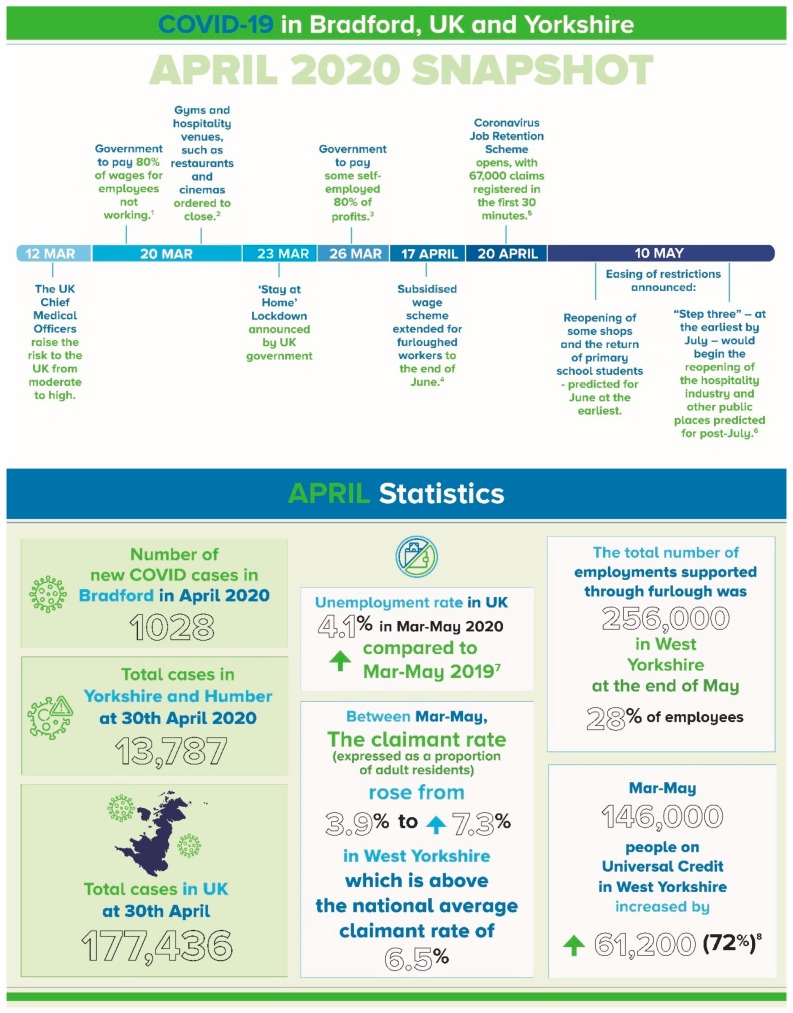
Timeline and Snapshot of COVID-19 events in Bradford, UK, as they affected the BiB participants. **Sources:** https://coronavirus.data.gov.uk/details/cases. ^1^ *“Wages cover for businesses hit by virus outlined”.* BBC News. 20 March 2020. ^2^ *“UK pubs and restaurants told to shut in virus fight”.* BBC News. 20 March 2020. ^3^ *“Coronavirus: UK government unveils aid for self-employed”*. BBC News. 26 March 2020. ^4^ *“Government extends furlough scheme to pay staff”.* BBC News. 17 April 2020—via bbc.co.uk. ^5^ *“Coronavirus: Millions to claim as UK furlough scheme goes live”*. BBC News. 20 April 2020. ^6^ *“PM address to the nation on coronavirus”.* GOV.UK. Prime Minister’s Office. 10 May 2020. ^7^ ons.gov.uk/employmentandlabourmarket/peoplenotinwork/unemployment. ^8^ westyorks-ca.gov.uk/media/4107/lcr-covid-19-monitor-19062020-final.pdf.

The qualitative findings presented here examine and compare the responses from mothers to open-ended questions about young families’ experiences during the initial stages of the global crisis, from these two distinct international centres. Figure 1 and Figure 2 provide timeline and key contextual information during the data collection period for both samples. While a small proportion of fathers completed the questionnaire from both cohorts, this paper focuses on the data collected from mothers. 

### 2.4. Participants

A total of 903 (400 for ORIGINS and 503 for BiB) participants with children aged 0–4 completed the two surveys during April 2020. Whilst the mean age of the parents completing the surveys was very similar (33.6 years old in ORGINS and 33.3 years old in BiB), there were differences in ethnicity, education and deprivation scores. The majority of the sample of BiB parents were of Pakistani heritage (57.7%) compared to 52% of the ORIGINS participants who identified their ethnicity as British/Irish. Over half of the respondents (55%) in ORIGINS had completed a bachelor or postgraduate degree, compared to 34.3% of BiB respondents. The characteristics of these sample populations are shown in Appendix A (for ORIGINS) and Appendix B (for BiB) Although a direct comparison of the deprivation scores cannot be made, Appendix A shows that the ORIGINS families are more likely to be clustered in less deprived areas, and Appendix B shows that the BiB families are more likely to be clustered in more deprived areas. 

## 3. Results

### 3.1. Biggest Worries

Despite these demographic differences, the main themes that emerged from the ORIGINS and BiB data were very similar. Where we found difference was in emphasis and levels of intensity. In both cases, the most commonly reported worries were: (a) Financial Insecurity & Employment; (b) COVID-19 Health Anxiety; (c) Educating and Caring for Children; (d) Current and Future Impact of COVID-19 on Society; (e) Child Development and Wellbeing; (f) Mental Health; and (g) Not Seeing Friends and Family. 

The ORIGINS and BiB parents both frequently commented they were worried about their household finances and money. Relatedly, there were worries regarding actual and potential changes to their own or their partner’s employment as a result of the pandemic. These are some examples (Table 1) from the ORGINS responses (*plain* italics) and BiB responses (***bold*** italics):

One difference that we identified from our comparison of the responses is that the ORGINS families seemed more likely to be worried about finances and job loss in the abstract, whereas for the BiB families it was more apparent that employment and family income had already become less secure due the lockdown.

*“Lack of income, husband is on furlough but is yet to receive any kind of payment. Child has special dietary needs and we are struggling to afford them right now. Very worried about how we will survive without regular income”*

*“Partner is not working don’t know when this pandemic will end, struggling with bills and stuff”*

*“My husband is a self-employed worker his role is a taxi driver. Since the outbreak of corona-virus it has affected us very bad. [It is] impossible to carry out a 2 metre social distance with passengers. It’s a big worry financially for us [we have] got bills and other direct debit payments.”*

Worries and anxiety regarding COVID-19 were also commonly reported, especially among the BiB cohort (Table 2). This included concern that themselves, immediate family members and extended family members (including those living overseas) might contract the virus and become seriously ill and/or die. 

This was a particular worry for people who were key workers or who had family members that were. 

Related to health anxiety were parents’ worries around mental health, both their own and that of their family and friends (Table 3). Some BiB respondents reported that the current situation had intensified their existing mental health problems, such as depression, anxiety, OCD and psychosis. More were feeling overwhelmed having to balance an increased amount of domestic labour alongside paid work, without any break or opportunities to leave the home and relax. 

These feelings were exacerbated by the social isolation of lockdown, being unable to access support and help with childcare, especially for new and/or single parents:

*“No family members to help feels helpless at times.”*

*“Not being able to socialise, difficult being at home when you are a new mum”*

Respondents also commented that they were worried about the development and wellbeing of their infant/child (Table 4). For many families, the lockdown meant their child was unable to attend their regular educational and social programs; parents expressed concern regarding the impact of this on their child’s development. 

Finally, respondents also raised concerns (Table 5) regarding both the current and future impact of COVID on society, including the state of the economy and how this will impact on their finances. 

### 3.2. Challenges

In response to the question “Tell us about a challenge you have faced in the last two weeks?”, the main themes were: (a) Educating and Caring for Children; (b) Financial Insecurity & Employment; (c) Missing Family and Friends; d) Family Relationships; (e) COVID-19 Health Anxiety; and (f) Non-COVID Health Concerns. 

The majority of responses centred on the theme of educating and caring for children while working from home and engaging in household tasks (Table 6). Mothers frequently reported that they were finding it difficult to balance work commitments and time for family. 

### 3.3. Enjoyable Aspects

In response to the question “Can you tell us how lockdown has made any parts of your life easier or more enjoyable?”, the main themes were: (a) Relaxed Routines; (b) Quality Time with Family; and (c) Positive Impact on Child Development. 

The majority of mothers reported lockdown had meant their usual routines were more relaxed and that they were able to spend more quality time (and in some cases more time outdoors) with their immediate family, including their partners who were at home more (Table 7). There were comments about spending to less time travelling or commuting for work and activities which allowed for more time with family. Those respondents who had recently had a baby reported they were enjoying the time with their newborn without having to be social or share their newborn with others. 

Some respondents reported that the lockdown had a positive impact on their child’s development (Table 8). 

Respondents in the ORIGINS cohort also reported that the lockdown had a positive impact on their household finances and families were able to save money that would typically be spent on activities and expenses outside the home: for example *“Spending less money on activities etc.”* and *“We’ve been able to save more”*. This type of response was much less common in the BiB sample.

Parents in both surveys also reported that they had not experienced any enjoyable aspects of lockdown, particularly for the BiB families (Table 9). 

## 4. Discussion

The focus of governments across the world during the COVID-19 pandemic has largely been on medical resources to treat those who contract the virus, and on restrictions to manage the spread of the virus. However, the emotional wellbeing, needs and concerns of whole populations facing acute restrictions and uncertainty must also be understood, managed and supported given the short and long-term implications for health. Our family cohort studies provided an important opportunity to document the impact on young families—in their own words—and look at differences and similarities experienced in two distinct international centres at the onset of a global crisis. 

Overall, despite varying in geography, ethnic background, levels of socio-economic disadvantage and the situational context during the pandemic (e.g., virus exposure and case numbers) the experiences of families from the ORIGINS and BiB were very similar. 

Respondents from both cohorts reported a number of worries and challenges during the lockdown period including employment/finances, health anxiety, mental health and social isolation, caring for children and child development. 

The most common and consistent themes were high levels of health anxiety respondents reported related to fear that they or family members would contract the virus. For the BiB cohort this reflected the reality of the situational context, but families in the ORIGINS cohort reported a similar concern related to a perceived threat of contracting the virus, even though community cases were much lower. 

Families across both cohorts also reported concerns regarding their child’s wellbeing and development. In particular, many were worried how their child’s development could be impacted by not attending regular educational and social programs. In addition, mothers commonly reported that they were finding it difficult to balance work commitments and time for family. This suggests that mothers are vulnerable to feeling the mental load of the demands of home schooling, childcare, domestic tasks and paid employment during the pandemic. 

Mothers in both cohorts also reported concerns regarding finances and employment, both worried about current changes to their employment and financial security and the perceived future threat to their employment and financial situation. ORGINS families were more likely to be worried about finances and potential job loss in the abstract, whereas many BiB families were already feeling the immediate and tangible effects of lockdown on their employment and financial situation. 

An advantage of this study is that both the ORIGINS and BiB surveys included open-ended questions regarding any perceived “positive” aspects of the lockdown. Again, respondents from both cohorts reported benefits from relaxed routines and increased time with family during the lockdown period. However, this was more evident in the ORIGINS cohort. This is consistent with other international reports early during the COVID-19 lockdowns, which identified a number of positive aspects of the pandemic-related changes—including spending more time with family [5,20,21], more opportunities to exercise, increased work flexibility and additional time to rest, which in turn provided an opportunity to reflect on priorities [5,18,19]. Studies have also reported an increase in kindness to others and people feeling more connected to their community [5,21,22]. 

## 5. Strengths and Limitations

Our study highlights the opportunities that existing cohort studies may provide in answering new challenges: firstly, as a network for collaboration and harmonisation of data collection, and secondly as a valuable resource of long-term data on the health and wellbeing of families. Cohort studies of young families provide the ability to track whole communities and identify common concerns in health and other social determinants, such as psychosocial, financial and educational. Importantly, this ability to monitor in real-time can indicate critical opportunities and ways to intervene to prevent future health problems in subsequent generations.

The use of qualitative methods was essential to capture the unique experiences of families during the COVID-19 lockdown through the exploration of themes in response to three open-ended questions. The relatively large sample size from both cohorts means it would not have been possible to collect the breadth of responses using individual in-depth qualitative interviews. Alternatively, closed question surveys would not have adequately capture young families’ experiences and priorities in their own words. However, a limitation of collecting this type of qualitative data is that some responses were short in length which meant the authors sometimes had to do more interpretation of participants’ meaning. This was addressed by having multiple researchers review the same responses in both sites to independently code the data before collaboratively identifying synergies and divergences in the responses.

This study captured the initial impact and reaction to the COVID-19 lockdown in April 2020. While conducted within a particular timeframe, this study was able to highlight the worries and priorities for young families in two different geographical locations during that unique period of time, rather than reflecting on this retrospectively. However, it should be noted that future studies are needed to compare and understand what has changed in terms of families’ experience and priorities since the beginning of the pandemic and the initial lockdown. This work should also consider how the impact and experience varies between the different COVID-19 contexts in UK and Australia

## 6. Future Surveys

Going forward, longitudinal data—both quantitative and qualitative—from ORIGINS and BiB will be critical in examining the long-term impact of the pandemic on the health and wellbeing of families in two international centres. For example, the routine data collected as part of the longitudinal follow-up with families enrolled in ORIGINS and BiB will enable future research to examine the impact of parental stress and anxiety on child wellbeing and development, as well as examining the impact of limited access to schooling, support services and socialisation on child development. 

Our cohorts are co-created in partnership with the communities they serve. It is critical to recognise the importance of all voices in shaping the future health narrative. Studies of this nature also provide an invaluable opportunity to capture the voices of the community in a meaningful way—and sharing experiences and learning between communities across the world. 

Further collaborative research between the ORIGINS and BiB cohorts is now underway to examine the attitudes and perceptions of the general population regarding the COVID-19 vaccination, as well as the intention to vaccinate among parents and children. This research will assist in understanding how geographical differences, levels of socio-economic disadvantage, trust in authority and the situational context impact on vaccination intentions and attitudes, and in turn how information about the COVID-19 vaccine needs to be communicated and marketed to families with young children. 

This collaboration has demonstrated that families across the globe are experiencing many shared positive and negative immediate impacts of COVID-19. This reinforces that, irrespective of situational context and differences in the actual level of threat, people demonstrate similar responses and needs in times of crisis. This population-based data has the potential to be used to underscore the need for support services, and inform the development of public health campaigns and universal interventions to assist families in future health crises. This research has shown that families across the general population need additional information and support during a pandemic to manage the immediate impact. Future research conducted through the ORIGINS and BiB cohort collaboration will be vital in determining the long-term impact of the pandemic and needs of families with young children. 

## 7. Conclusions

In summary, the key points from this research include:

- Families across the globe experienced both positive and negative immediate impacts of COVID-19
- Cohort studies of young families provide the ability to track whole communities and identify common concerns
- Population-based data can be used to inform the development of support services, public health campaigns and universal interventions to assist families in future health crises
- Globally, cohort studies provide a network for collaboration and harmonisation of data collection, research opportunities and knowledge translation.

## Figures and Tables

**Table 1:** Financial concerns: Sample responses.

**ORIGINS**	*“The economy and how it will affect us financially”* *“Not being able to pay rent and bills”* *“That my partner will lose his job (almost happened last week)”*
**BiB**	***“We won’t be able to cope financially”*** ***“That’ll we will get in even more debt”*** ***“Financial stability, can I be able to live under lock down and support my family”***

**Table 2:** Fear for family safety: Sample responses.

**ORIGINS**	*“That someone I love is going to get seriously ill”* *“Bringing COVID home to my family”*
**BiB**	***“That one of my family members that is a key worker will catch the virus”*** ***“Losing people I care about”***

**Table 3:** Mental health/social isolation: Sample responses.

**ORIGINS**	*“Mental health and wellbeing-no contact from friends or family and kids not going to school makes all of us feel a little down about life”* *“Lack of social connections for kids and me”* *“Not being able to see family and friends”* *“Home schooling while managing a toddler and all other home duties”*
**BiB**	***“Having a nervous breakdown or a panic attack…can’t get a break from all the responsibilities and go somewhere for fresh air even”*** ***“Balancing all our responsibilities-home schooling/going into work/working from home/housework-Feeling stressed”*** ***“How am I going to manage if this goes on any longer”*** ***“Feeling suffocated”*** ***“Hurting myself because my depression/anxiety take a turn for worse”*** ***“My partner is a key worker and has had to go sick leave due to his anxiety”***

**Table 4:** Concerns about children’s education and behaviour: Sample responses.

**ORIGINS**	*“My son has GDD (Global Development Delay) and* *has challenging behaviours. Worry about him not getting* *the support he needs as structured activities have stopped for him”* *“Child becoming more clingy and co-dependent because* *his world has shrunk (currently only seeing people in 2 households)”*
**BiB**	***“Keeping my two year old entertained all day as she doesn’t*** ***understand why she can’t go out to toddler groups/*** ***swimming and to see family etc.”*** ***“Worried about daughters learning and development as she only recently started nursery and was starting to develop but going back to her pre-nursery phase now”***

**Table 5:** Long term concerns: Sample responses.

**ORIGINS**	*“That life as we know it has changed forever”* *“This virus never getting better or going away and* *living like this for a long time”* *“If the state of the economy will make it harder* *to buy a house (we had planned to buy at the end of this year)”*
**BiB**	***“How coronavirus will affect us in the long*** ***term such as the economy, socialising, etc.?”*** ***“How is the lockdown going to affect things long*** ***term i.e., education and society in general”*** ***“the future and the wider impact of the*** ***virus on my family and wider society”***

**Table 6:** Tasks and responsibilities: Sample responses.

**ORIGINS**	*“Hard to home school, work and do the household * *chores all at the same time”* *“Balancing work and the kids and not * *having time to debrief/think about the day”* *“Finding the time to work, teach my daughter, clean, cook”*
**BiB**	***“Cooking, cleaning, trying to please everyone”*** ***“Everything feels like a challenge, ensuring *** ***the kids are fit and healthy and well-fed”*** ***“Teaching my kids school work and balancing *** ***housework as well as fitting in some me time”***

**Table 7:** Enjoyable aspects: Sample responses.

**ORIGINS**	*“Knowing you can’t go anywhere has slowed life down a bit* *and nice to not be rushing and having time with my children”* *“I am quite enjoying the slowed down pace and being less* *stressed with things to do and places to be”.* *“I actually am enjoying being home as a family unit more.* *We have all grown closer but it can be challenging* *not to get irritated by one another”.* *“Spending quality time with my children”* *“Less travel for work. More time at home with my mum and girls”* *“Being able to enjoy our newborn just to ourselves”*
**BiB**	***“Spending and enjoying time with my children and *** ***not rushing to work. If anything it work out better*** ***for me as I’ve been really enjoying been with my children and just having more family time which I LOVE!”*** ***“My husband is home all the time and I’m loving it, *** ***we do everything together”*** ***“More family time and less rushing around. *** ***Better work life balance”***

**Table 8:** Positive impacts: Sample responses.

**ORIGINS**	*“Toilet training my toddler has been easier while we are home more!”* *“5 yr old can ride her bike now”*
**BiB**	***“I love my children all together and playing together. My two year old is so much happier and entertained with his brothers and sister at home. He’s learnt so many new words and his development has advanced so much in just a couple of weeks”***

**Table 9:** Negative impacts: Sample responses.

**ORIGINS**	*“I don’t feel that any aspects are easier at the moment”*
**BiB**	***“it has not [been enjoyable], made it a lot harder”*** ***“if I wasn’t struggling then I would enjoy this time more. I always worry what my kids will eat next”***

## Data Availability

Details of how to access BiB data or collaborate with BiB can be found online https://borninbradford.nhs.uk/research/how-to-access-data/ (accessed on 23 August 2021) For queries and details relating to accessing ORIGINS data, please email: origins.research@telethonkids.org.au.

## Appendix A. Characteristics of the ORIGINS Survey Sample (N = 400)

	**Mean (Standard Deviation)**
**Age: n = 400**	33.6 (4.7)
	**n (%)**
**Currently Pregnant: n = 400**	
No	333 (83.2%)
Yes	67 (16.8%)
**Pregnant and first time mother: n = 389**	
No	362 (93.1%)
Yes	27 (6.9%)
**Adults living in house: n = 389**	
1	5 (1.3%)
2	355 (91.3%)
3	20 (5.1%)
4	7 (1.8%)
5	1 (0.3%)
6	0 (0%)
7	1 (0.3%)
**Children living in house: n = 389**	
0	27 (6.9%)
1	189(48.6%)
2	115 (29.6%)
3	45 (11.6%)
4	10 (2.6%)
5	3 (0.8%)
**Education: n = 361**	
Year 10	17 (4.7%)
Year 12	45 (12.5%)
Trade	42 (11.6%)
Bachelor degree	136 (37.7%)
Post Graduate degree	86 (23.8%)
Other	35 (9.7%)
**Ethnicity: n = 342**	
Australian	69 (20.2%)
New Zealand	8 (2.4%)
British/Irish	178 (52.0%)
European	46 (13.5%)
North African or Middle Eastern	3 (0.8%)
Asian	30 (8.8%)
American	6 (1.8%)
African	2 (0.5%)
**Socioeconomic status** **SEIFA IRSAD (deciles): n = 400**	
1	1 (0.3%)
2	19 (4.8%)
3	3 (0.8%)
4	13 (3.3%)
5	10 (2.5%)
6	52 (13.0%)
7	39 (9.8%)
8	101 (25.3%)
9	101 (25.3%)
10	61 (15.3%)

## Appendix B. Characteristics of the BiB Survey Sample (N = 503)

	**Mean (Standard Deviation**)
**Age: n = 489**	33.3 (5.7)
	**n (%)**
**No. of children living in house: n = 503**	
1	78 (15.6%)
2	132 (26.2%)
3	153 (30.4%)
4	88 (17.5%)
5+	52 (10.4%)
**Education: n = 440**	
No qualifications	25 (5.7%)
<5 GCSE (grades A–C) or equivalent	105 (23.9%)
5 or more GCSE (grades A–C) or equivalent	66 (15.0%)
A levels or equivalent	82 (18.6%)
Degree or equivalent	151 (34.3%)
Don’t know	6 (1.5%)
Other	5 (1.1%)
**Ethnicity: n = 477**	
Asian or Asian British-Pakistani	275 (57.7%)
White British	122 (25.5%)
Other	80 (16.8%)
**Index of Multiple Deprivation: n = 488**	
1	321 (65.8%)
2	77 (15.8%)
3	46 (9.3%)
4	10 (2.1%)
5	7 (1.4%)
6	10 (2.1%)
7	7 (1.4%)
8	7 (1.4%)
9	3 (0.6%)
